# RESTRICTED YET EXPANDED: EVOLVING MOBILITY OF OLDER ADULTS WITH COGNITIVE IMPAIRMENT IN URBAN CHINA

**DOI:** 10.1093/geroni/igae098.2814

**Published:** 2024-12-31

**Authors:** Qingqing Yin, Lin Chen

**Affiliations:** University of Pittsburgh, Pittsburgh, Pennsylvania, United States; Fudan University, Somerville, Massachusetts, United States

## Abstract

Cognitive impairment restricts older adults’ mobility within and beyond the neighborhood. Existing literature primarily focuses on the declining mobility of older adults with mild cognitive impairment (MCI) and the implications of such decline for this vulnerable group. However, how older adults with MCI experience their changing mobility has received limited attention. Informed by community gerontology and the preventive and corrective proactivity (PCP) model, this study aims to explore how older adults with MCI perceive and adapt to their changing mobility. Through purposive sampling, we took a phenomenological approach to conduct face-to-face, in-depth interviews with community-dwelling older adults with MCI in Zhengzhou, China (N = 34). By applying the geographic information systems (GIS), we spatially situated participants’ routine activities to visualize their changing mobility. Recognizing their cognitive decline, most participants voluntarily constrained their out-of-home activities within the neighborhood. Still, they tried to keep a strong grip on the existing mobility and prolong independent living as much as possible. One of the most useful strategies was to take advantage of their social networks in the neighborhood. Social interactions and mutual support created new opportunities for participants, which facilitated their out-of-home mobility even beyond the neighborhood. Their restricted mobility in the physical environment was thus continually defended and expanded by the social environment. The findings of this study inform the importance of promoting neighborhood built and social features to support older adults with different levels of cognitive and mobility functioning.

